# Gender trends in authorship of randomized clinical trials in dentistry

**DOI:** 10.1590/1807-3107bor-2025.vol39.082

**Published:** 2025-09-08

**Authors:** Mayara Colpo PRADO, Lara DOTTO, Bernardo Antônio AGOSTINI, Rafael Sarkis ONOFRE

**Affiliations:** (a) Atitus Educação, Graduate Program in Dentistry, Passo Fundo, RS, Brazil.; (b) Atitus Educação, Undegraduate Program in Dentistry, Passo Fundo, RS, Brazil.; (c) Atitus Educação, Graduate Program in Dentistry, Passo Fundo, RS, Brazil.

**Keywords:** Authorship, Evidence-Based Dentistry, Dentistry, Gender Equity, Randomized Controlled Trial

## Abstract

The aim of the study was to explore female authorship in various aspects of randomized controlled trials (RCTs) in dentistry. A search was performed in PubMed for RCTs, in dentistry, indexed from 12/31/2016 to 12/31/2021. Only studies in English were considered. Data selection and extraction were performed by two authors and the following data collected: year of publication, journal, subject, number and names of authors, and country and gender (Genderize website) of the first 10 authors. Descriptive analyses, graphs, and maps were generated. Poisson regression assessed the influence of continent and year of publication on the presence of women as first or last authors. The results were presented as prevalence ratios (PR) and 95% confidence intervals (95%CI). A total of 844 RCTs and 4,305 authors (2,372 men and 1,662 women) were included. Gender disparity increases as the order of authorship advances. Among first authors, men represent 50.59% and women 44.08%, whereas among last authors, they account for 61.92% and 34.03%, respectively. Analyses showed no association between year of publication and the presence of women as authors. There were fewer women as first authors in Europe (PR: 0.82, 95%CI: 0.68–0.99) and as last authors in Europe and Asia (PR: 0.68, 95%CI: 0.53–0.87 and PR: 0.79, 95%CI: 0.63–0.99, respectively). The findings highlight a lower presence of female authors in all aspects analyzed in the RCTs, especially in last authorship. Also, there has been no indication of improvement in recent years. Female participation in RCTs is crucial not only for gender equity but also as a means to enhance the quality and relevance of clinical data for decision-making.

## Introduction

Gender equity is a fundamental right and a crucial determinant of health and economic development within society.^1,2^ Achieving this equity is one of the 17 sustainable development goals (SDGs) established by the United Nations in 2015, with a target for accomplishment by 2030.^3^ However, the latest report on SDG progress presents a troubling outlook. Despite being over halfway to 2030, progress toward gender equality falls significantly below expectations, with no goal indicators reaching the “target met or almost met” threshold.^4^

Gender disparities persist across nearly all sectors of society, including dentistry. Unequal gender representation may restrain the profession’s development and performance.^5,6^ Besides fostering team well-being, equity promotes the recruitment and retention of top talent. Consequently, it stimulates innovation, productivity, and the advancement of clinical, educational, and scientific efforts.^2,7-9^ Moreover, a more diverse team can produce more pertinent, applicable, and advantageous research to a wider population.^2^ Thus, ensuring gender equity and enacting policies to tackle the underrepresentation of women in specific dental sectors can serve as pathways to enhancing oral health for the population.^5,6,10^

In dentistry, gender differences are particularly noticeable in scientific research and leadership positions.^5,9,11^ Advancement within the field of science, often a catalyst for professional leadership, heavily relies on individuals’ academic activity, notably research article publications.^12^ Nevertheless, studies suggest that women are underrepresented in research authorship, thus being hindered from achieving an equitable distribution of recognition, opportunities, and positions.^11,13^ This scenario can be even more challenging in the context of randomized clinical trials (RCTs). While these studies are considered the foundation for clinical decision-making, they are also known for their high financial costs and substantial demands on time and resources. These factors can pose an additional barrier to the development of RCTs, particularly for women, as previous studies have indicated that they receive fewer incentives and have less access to research funding.^14-17^

Understanding the extent and locations of these inequities within dental science is essential for effectively addressing them. Hence, the objective of this study was to explore female authorship within a sample of RCTs in dentistry through various aspects, such as order of authorship, year of publication, country, and specialty of the RCT.

## Methods

The findings of this study were reported in accordance with the Sex and Gender Equity in Research (SAGER) guidelines.^18^ The protocol for this meta-research study is publicly available on the Open Science Framework platform.

### Sample

This sample was derived from a larger project aimed at mapping RCTs in dentistry, as detailed in the protocol mentioned above and in a previous publication.^19^Because of the large number of RCTs published annually (more than 500 RCTs according to Sarkis-Onofre et al.^20^), the sample size was calculated considering a 5% error probability (α = 0.05), an 80% power (1-β), an equal proportion of exposed and unexposed (women and men), and the estimated effect size of odds ratio (OR) = 1.5 based on a previous study with female team contribution.^21^ The OpenEpi software^22^ was used for this analysis, resulting in the inclusion of 844 RCTs.

**Figure 1:**
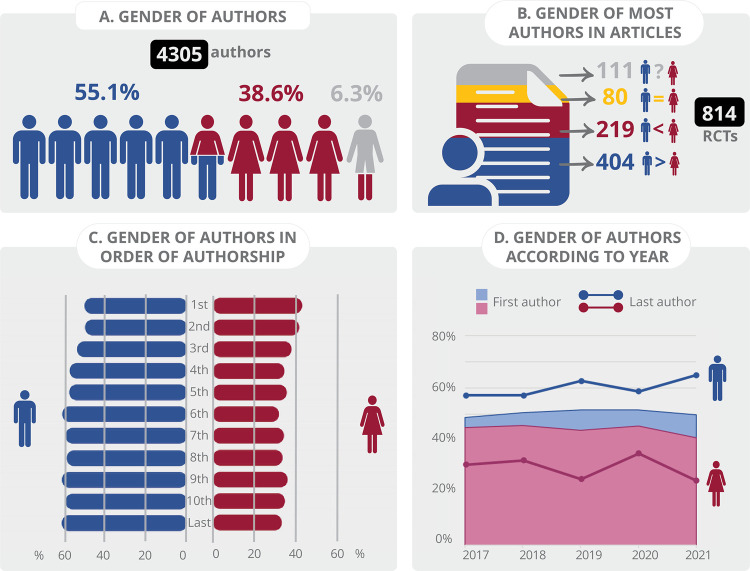
Gender distribution across various aspects: A) Total number of authors; B) Majority of authors involved in each RCT; C) Order of authorship; and D) Year of publication of the RCTs. Studies with more than 10 authors were excluded from analyses requiring the evaluation of all authors or the last author. Blue represents male authors or studies with a majority of male authors. Red denotes female authors or studies with a majority of female authors. Gray indicates authors of undetermined gender or studies in which the gender majority could not be determined due to unknown gender of one or more authors. Yellow represents studies with an equal number of male and female authors.

The search for studies was carried out in PubMed using a strategy developed by the authors based on MeSH terms and limited to the period from December 31, 2016, to December 31, 2021, which corresponded to the last 5 years prior to the completion of the study protocol. The full search strategy can be found in the Figure 1. The inclusion of RCTs, as described by Friedman,^23^ was limited to the dental field, regardless of the issues addressed, methods, or level of study reporting. Studies in languages other than English were excluded.

Screening for study inclusion was conducted using DistillerSR software (Evidence Partners Incorporated, Ontario, CA) based on a form that had been previously tested on 20 articles. Two reviewers (MCP and LD) independently screened all titles and abstracts and classified them as ‘‘include,’’ ‘‘exclude,’’ or ‘‘uncertain.” Full articles classified as included and uncertain were selected for further eligibility screening by the same two independent reviewers. Discrepancies in the selection of titles/abstracts and full articles were resolved through discussion or with the input of a third reviewer (RSO) in case of disagreement. The selection of the 844 studies was conducted using a random number list generated in Excel, based on the studies that passed additional eligibility screening. The proportion of indexed articles per year was considered when selecting the 844 RCTs.

### Data extraction

A pilot test was conducted through discussion between the three reviewers (MCP, LD and RSO) to test the extraction form and ensure consistency in the interpretation of the items. The pilot test included 20 studies randomly selected through a random number table generated in Excel. Data from the 20 studies included in the pilot test were re-extracted during the final data extraction. Data were extracted from the 844 RCTs using the DistillerSR online software (Evidence Partners Incorporated, Ontario, Canada). Two reviewers (MCP and LD) extracted data from half of the included studies each, while another reviewer (RSO) verified the consistency of data extraction and interpretation. In cases of doubt or inconsistency, data were re-extracted by the two reviewers who collected the data (MCP and LD). The following data were collected: year of publication, journal, article subject, number of authors and country, names and genders of the first 10 authors. Genders were determined by entering each author’s first name into the Genderize database. In cases in which gender could not be strongly inferred (90% probability threshold, based on Franco et al.),^24^ attempts were made to identify it through institutional websites or platforms such as ResearchGate.

**Table:** Top five journals in which men and women most publish RCTs as first and last authors.

Top 5	First author			Last author			
	Male		Female	Male		Female	
	Journal	n	Journal	Journal	n	Journal	n
1	Clinical Oral Investigations	29	Clinical Oral Investigations	Clinical Oral Investigations	39	Clinical Oral Investigations	23
2	Clinical Oral Implants Research	28	Journal of Dentistry	Clinical Oral Implants Research	28	American Journal of Orthodontics and Dentofacial Orthopedics	9
3	Clinical Implant Dentistry and Related Research	22	BMC Oral Health	Clinical Implant Dentistry and Related Research	23	Photodiagnosis and Photodynamic Therapy	8
4	Journal of Oral and Maxillofacial Surgery	20	Journal of Clinical Periodontology	BMC Oral Health	19	Journal of Dentistry	7
5	Journal of Clinical Periodontology	14	International Journal of Dental Hygiene	Journal of Clinical Periodontology	16	Journal of Clinical Periodontology	7

The subjects were classified in alphabetical order.

### Data analysis

Descriptive analyses were conducted using Microsoft Excel. Graphs were built to show the total number of authors by gender, the predominant gender among authors in each study, and the gender of authors by authorship order. Additional graphs were developed to represent the gender of the first and last authors by year, by continent, and the gender of the first author by article specialty. The top five journals in which men and women predominantly published RCTs as first authors were identified. Additionally, a map representing the number of women in the first and last authorship positions, considering the country of affiliation provided by the authors, was prepared. The darker the color of a country on the map, the more women in first or last authorship were present in that country. Studies with more than 10 authors were disregarded in analyses requiring the evaluation of all authors or the last author.

Stata 14.0 software (Stata Corp., College Station, USA) was used to perform Poisson regression analysis with robust variance to assess the influence of the author’s continent and the year of publication on the presence of females as first or last authors. The outcome was the female representation in these authorship positions. Prevalence ratios (PR) and 95% confidence intervals (95%CI) were used to present the results, with significant differences considered at p-values < 0.05. We used the PR because, in cross-sectional studies, the OR is often overestimated, and the PR provides a more precise and reasonable measure of effect.^25,26^ The tested hypotheses were: a) female representation as first and last authors may vary across continents due to differences in inclusion policies among countries, and b) the proportion of female authorship may have changed over the analyzed time period.

## Results

A total of 4,305 authors were identified in the 814 studies with 10 or fewer authors, comprising 2,372 men (55.10%) and 1,662 women (38.60%). The remaining 271 authors (6.30%) had indeterminate gender, according to the Genderize database (Figure 1A). Among the analyzed articles, 404 articles (49.63%) were predominantly authored by men, 219 (26.90%) were predominantly authored by women, and 80 articles (9.83%) had an equal representation of both genders. In 111 articles (13.64%), it was impossible to define whether most authors were men or women or whether there was no gender difference because the gender of one or more authors could not be identified (Figure 1B).

Figure 1C shows the gender of authors by authorship order. We observed the smallest difference in the first two authorship positions with 50.59% men and 44.08% women filling the first place and 50.36% men and 42.60% women found in the second place. The gender gap widened further, reaching a difference of over 29% in the sixth position (61.65% men and 32.58% women). The difference remained substantial in the subsequent authorship positions up to the last authorship position with 61.92% male authors, compared to 34.03% female authors. Overall, male researchers prevailed, regardless of authorship position.

Figure 1D demonstrates that regardless of authorship position, men seem to consistently outnumber women in all authorship positions over the years. Yet, first authorship is systematically more prevalent among women, while last authorship is more prevalent among men. Another important aspect is that the proportion of men seems to remain relatively constant for first authorship from 2017 to 2021 (a variation of 2.87%), while the percentage of women varied from 44.88% in 2017 to 41.03% in 2021. In contrast, the percentage of men as last authors rose from 59.02% in 2017 to 65.95% in 2021. The proportion of women shows an irregular pattern between the years (a variation of 9.35%), ending with a lower proportion in 2021 (29.73%) compared to 2017 (35.24%). The regression analysis, however, did not demonstrate an association between year of publication and female presence in either the first or last author positions.

**Figure 2:**
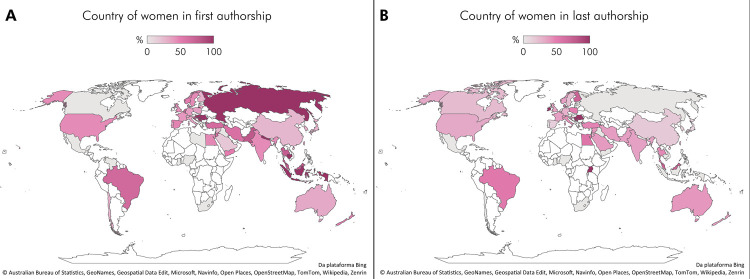
Map representing the distribution by country of women in first (A) and last authorship (B) of RCTs.

Regarding the distribution of women as first and last authors of RCTs by country (Figure 2), the map for first authorship (A) shows darker colors compared to the map for last authorship (B), indicating more countries worldwide with a considerable number of women as first authors of RCTs. Countries such as Russia and Indonesia reached 100% female representation as first authors of RCTs and 0% as last authors. The opposite pattern (100% as last authors and 0% as first authors) was observed in countries such as Uganda, Serbia, and Ireland. However, these are countries with only a few or a single RCT attributed to them. Countries with a higher number of RCTs, such as Brazil, China, Germany, India, and the United Kingdom, maintained a similar percentage of women as both first and last authors. One of the largest declines in the prevalence of women as first and last authors was observed in the Netherlands, while Denmark showed the largest increase.

The assessment of authorship by continent revealed that in all continents, except for Africa, there are more women as first authors than as last authors, with the smallest difference observed in Oceania and the largest one in America. The statistical analysis showed, a lower proportion of women as first authors in Europe (PR: 0.82, 95%CI: 0.68–0.99) when compared to the Americas, and a lower proportion as last authors in both Europe and Asia (PR: 0.68, 95%CI: 0.53–0.87 and PR: 0.79, 95%CI: 0.63–0.99, respectively).

Table shows the five journals in which men and women publish most RCTs as first and last authors. There is a predominance of journals in specific areas when men are the first authors, such as surgery and implants. Only one journal has changed in terms of males as last authors (Journal of Oral and Maxillofacial Surgery for BMC Oral Health). On the other hand, the journals in which women publish more RCTs as first authors are more general and can cover different areas of dentistry. Journals change considerably when women as last authors are evaluated.

**Figure 3:**
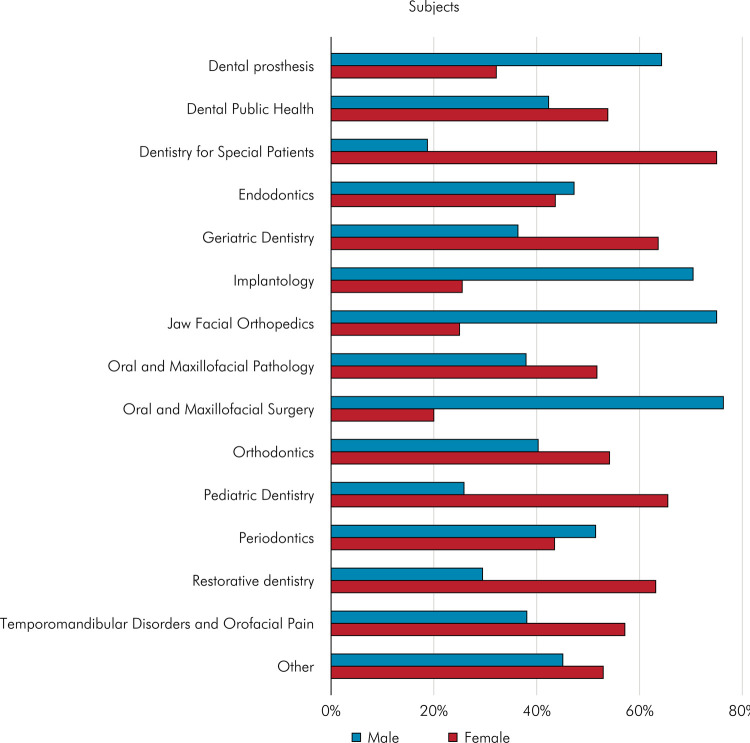
Gender of the first author according to the dental subject of the articles.

Figure 3 presents the gender of the first author according to the subject of the article. There are specialties in which one gender predominates over the other. Subjects such as oral and maxillofacial surgery (76.30%), jaw facial orthopedics (75.00%), implantology (70.41%), and dental prosthesis (64.29%) are predominantly chosen by men as first authors. Conversely, dentistry for special patients (75.00%), pediatric dentistry (65.52%), geriatric dentistry (63.64%), and restorative dentistry (63.16%) are areas preferred by women as first authors. The following link provides the sample n and the percentage for all results presented in the figures.

## Discussion

This is the first study exploring different aspects of female authorship in RCTs, a type of clinical research extremely important in evidence-based practice. Our findings reinforce that although there have been many advances in gender equity regarding the number of women in undergraduate studies and the job market, this is still not observed in the authorship of scientific articles over a five-year period (2017–2021). Unfortunately, the results indicate a lower proportion of women authors than men in our sample and in practically all analyzed aspects. Particularly concerning are the findings regarding last authorship, which traditionally represents seniority and leadership, showing no prospects of gender equity improvement, considering the results presented over the past few years. These results underscore the urgent need for evidence-based measures to mitigate, at least, the inequalities faced by women in the profession.

The results for last authorship are concerning, with an important difference in the proportion of men (61.92%) and women (34.03%). The present results endorse the evidence about the lack of women leading research centers. Sartori et al.^13^ demonstrated that women represented only 22% of last authors in leading dental journals. However, it is important to note that: a) this study analyzed articles published in earlier years (2006, 2011, and 2016) and over a longer period than ours; b) although it included different study designs, it was limited to the 10 journals with the highest impact factor in each dental specialty; and c) author gender was initially determined using PubMed, Scopus, ResearchGate, and their respective affiliated institutions.^13^ Previous studies in dentistry and other fields worldwide have highlighted the underrepresentation of women in leadership roles in research.^2,5,9^

The challenges faced by women, which can be considered invisible barriers hindering their progress to higher-prestige positions compared to men, are numerous.^27-30^There are indications that the disparity between men and women in scientific research follows a cycle: at a certain point in their careers, men manage to publish more articles, which may be due to social and family issues that disproportionately burden women with domestic and childcare responsibilities, for example. A higher number of published articles, in turn, leads to greater incentives, scholarships, funding, invitations, and recognition for men. Therefore, men are encouraged to produce more scientific articles, perpetuating the cycle and preventing women from achieving parity.^31,32^

The pattern in first authorship, positions typically held by researchers in the early stages of their academic careers, remains unchanged. Smaller differences were observed between men and women. Therefore, one might suggest that the increasing number of women in dental education and the workforce is beginning to impact the academia, with potential future effects on last authorship. Previous studies have suggested this trend, showing a rising proportion of articles authored by women over time, increasing from 6% to 21% between 1985 and 2008 in a sample of authors associated with US institutions^27^ and from 26% to 33% between 1996 and 2015 in various types of dental studies obtained from the Scopus database.^11^ However, in our study, which examines the evolution of first and last author percentages between 2017 and 2021, we cannot confirm a similar growth. On the contrary, our statistical analysis indicates no significant trend of change in gender disparity over these years.

Note that our analysis covered a more recent period, in which the impact of women being the majority in dental education and the workforce around the world may have a greater influence on the findings, as mentioned above. Furthermore, our study covered a five-year period, which is shorter than the 20 years or more evaluated in previous studies.^11,27^The “leaky pipeline effect” in sciences, in which female scientists or academics tend to progress less or more slowly than men, may explain our findings.^33^ Thus, the analyzed period might be insufficient to observe a more significant evolution in the proportion of women in study authorship, which progresses slowly. Despite the goals set by various organizations to reduce gender inequalities,^3^ any changes, if they exist, are inadequate and progressing slowly. Moreover, we may have reached a plateau in women’s participation in science, with most women opting for other professional paths, especially in health sciences, in which the care or clinical market could be more profitable. Another possible explanation could be that the Genderize database is unable to accurately identify the gender of certain names, leaving around 6% of the sample with an indeterminate gender. Although these 6% would not fully equalize the number of women and men, they would reduce the difference, as noted in previous studies.^11,27^ This fact could be pointed out as a limitation of our study.

In our research, we identified a significantly lower proportion of female first authors in Europe and female last authors in both Europe and Asia compared to those in the Americas. Other studies have also shown that the proportion of female authorship in the Americas, particularly Latin America, is higher than in other regions around the globe.^9,13^ This can be explained by the high percentage of female researchers in Latin America (44%), driven by countries such as Brazil (55%), compared to Europe (33%).^9^ Political, economic, and cultural factors, along with social norms, may influence the results of our study and those in the literature, as well as the efforts made in different regions to integrate women into research and authorship roles.

We observed a more significant proportion of female first authors in specialty articles such as pediatric and geriatric dentistry. In contrast, surgery and implant dentistry articles were more frequently attributed to male first authors. This trend was also evident across the journals in which these articles were published. Once again, societal norms and gender expectations may account for specific specialties and journals favoring one gender. The underlying notion persists that women are inclined to take on caregiver roles, either in clinical or maternal capacities, which may justify a smaller number of publications. Conversely, men are often perceived as excelling in more invasive fields, with their sole responsibility being total immersion in work, whether technical or scientific.^6^ This dynamic is apparent not only seen in dentistry but across various professions. Previous research has indicated that women are discouraged from pursuing specialties with higher patient mortality risks and longer work hours, such as surgery.^34,35^ Furthermore, apprehensions about facing heightened discrimination in male-dominated professions may steer women towards specialties in which they are better represented.

It is important to note that our findings are based on a sample comprising a single study design — RCTs — in the dental field. In medicine, women were significantly less likely to be first or last authors of rheumatology RCTs published in high-impact journals between 2015 and 2019 compared to other research designs.^36^An even lower prevalence than that found in our study was observed in the authorship of case report studies in general medicine, 36% for first authorship and 25% for last authorship.^21^ In dentistry, the highest prevalence of woman first and last authors was found in epidemiological studies (40.0% and 31.9%, respectively), while the lowest prevalence was observed in review studies (31.2% and 21.2%, respectively).^13^ Also in dentistry, articles published in the three major orthodontic journals between 2008 and 2010 and 2018 and 2020 showed a lower proportion of women as first authors in in vitro studies (29.1%) and as last authors in meta-research studies (13.3%).^37^

Our study has some limitations. One of them concerns how gender was assessed, relying solely on the names provided by authors in the articles and adopting a binary classification (female or male) as per the categorization provided by the utilized website. Consequently, we could not evaluate gender within a broader contextual framework, as would be ideal. Furthermore, we were unable to determine the gender of all authors in studies with more than 10 authors (30 studies, approximately 3.5% of the sample); however, we believe that these data do not alter the general trend observed in our study. It is important to note that the scientific output of RCTs varies across countries. For instance, Russia, South Africa, and Indonesia had only one RCT attributed to them, which yields extreme percentages of female first and last authorship (0% in the absence of female authorship and 100% in its presence). Additionally, as mentioned previously, the sample was derived from a prior study that conducted a global mapping of RCTs, drawn from a single database, and exclusively included articles published in English. These last three factors may constrain the generalizability of our findings.

RCTs provide the highest quality primary evidence for health decision-making and are therefore essential for advancing dentistry.^23^ However, their high financial costs, along with the time and personnel investment required, may limit female authorship in these studies.^15,38,39^ Considering these factors and their importance in evidence-based practice, examining the proportion of women among RCT authors is valuable for identifying gender trends in dental science. The presence of female authors in RCTs can diversify research topics, particularly by highlighting health issues that exclusively affect women and/or have been overlooked in research predominantly conducted by men.^2,40^Some studies also suggest that the presence of female authorship in RCTs may influence the choice of more ethical methodologies or approaches, including the participation of women in sample groups, leading in greater diversity and relevance of data to real-world populations.^40^ Moreover, a higher proportion of female authors has been associated with a lower risk of bias in studies.^41^ Therefore, female participation in RCTs is not only crucial for gender equity but also for enhancing the quality and relevance of clinical data.

Publicly documenting disparities, in addition to acknowledging the issue, provides a means to track progress toward gender equality over time. Through the data generated and future research, exploring gender issues in various contexts, studies, and professions, steps must be taken to pursue the fundamental right of gender equity. The adoption of change strategies by governments, organizations, leaders, researchers, and society at large can foster gender equity not only within the profession but also comprehensively and globally.

## Conclusion

Our findings reveal that women are in a lower proportion than men in all authorship positions in RCTs, even after progressing in numbers in undergraduate and in the job market. The data show an important gender disparity in last authorship, one of the most prestigious positions that generally reflects research leadership. Furthermore, we found fewer women in first authorship in Europe and in last authorship in Europe and Asia. When women are first authors, they tend to publish in broader journals and in areas such as dentistry for special patients, pediatric dentistry, and geriatric dentistry. In our evaluation over time, we did not observe signs of a reduction in the gender gap in first and last authorship, suggesting that it is essential to accelerate the pace and propose more and better measures to promote gender equality in the profession.
